# Technical considerations of multi-parametric tissue outcome prediction methods in acute ischemic stroke patients

**DOI:** 10.1038/s41598-019-49460-y

**Published:** 2019-09-13

**Authors:** Anthony J. Winder, Susanne Siemonsen, Fabian Flottmann, Götz Thomalla, Jens Fiehler, Nils D. Forkert

**Affiliations:** 1 Department of Radiology, University of Calgary, Calgary, Canada; 2 Department of Diagnostic and Interventional Neuroradiology, University Medical Center Hamburg-Eppendorf, Hamburg, Germany; 3 Department of Neurology, University Medical Center Hamburg-Eppendorf, Hamburg, Germany; 4 Department of Clinical Neurosciences, University of Calgary, Calgary, Canada; 5 Hotchkiss Brain Institute, University of Calgary, Calgary, Canada; 6 Alberta Children’s Hospital Research Institute, University of Calgary, Calgary, Canada

**Keywords:** Brain, Predictive markers

## Abstract

Decisions regarding acute stroke treatment rely heavily on imaging, but interpretation can be difficult for physicians. Machine learning methods can assist clinicians by providing tissue outcome predictions for different treatment approaches based on acute multi-parametric imaging. To produce such clinically viable machine learning models, factors such as classifier choice, data normalization, and data balancing must be considered. This study gives comprehensive consideration to these factors by comparing the agreement of voxel-based tissue outcome predictions using acute imaging and clinical parameters with manual lesion segmentations derived from follow-up imaging. This study considers random decision forest, generalized linear model, and k-nearest-neighbor machine learning classifiers in conjunction with three data normalization approaches (non-normalized, relative to contralateral hemisphere, and relative to contralateral VOI), and two data balancing strategies (full dataset and stratified subsampling). These classifier settings were evaluated based on 90 MRI datasets from acute ischemic stroke patients. Distinction was made between patients recanalized using intraarterial and intravenous methods, as well as those without successful recanalization. For primary quantitative comparison, the Dice metric was computed for each voxel-based tissue outcome prediction and its corresponding follow-up lesion segmentation. It was found that the random forest classifier outperformed the generalized linear model and the k-nearest-neighbor classifier, that normalization did not improve the Dice score of the lesion outcome predictions, and that the models generated lesion outcome predictions with higher Dice scores when trained with balanced datasets. No significant difference was found between the treatment groups (intraarterial vs intravenous) regarding the Dice score of the tissue outcome predictions.

## Introduction

Due to the overwhelming success of stent retrieval devices in recent randomized controlled trials^1–5^, which was further confirmed in a pooled meta-analysis^6^, guidelines for the treatment of stroke patients with proximal arterial occlusions now recommend administration of intraarterial (IA) mechanical thrombectomy in conjunction with standard intravenous (IV) recombinant tissue plasminogen activator (rtPA)^7,8^. Careful patient selection is essential for maximizing the therapeutic benefit of thrombectomy while minimizing treatment cost. For example, the estimated thrombectomy cost, including additional time and specialized resources required, is estimated approximately four times that of IV rtPA alone^9^. The question if thrombectomy is associated with an increased risk of intra-procedural and post-operative complications^10^ is an area of active research. Although thrombectomy is generally reported to be safe^6,11^, an increased risk of intracerebral hemorrhage has been reported specifically for patients with large (ASPECTS <6) infarcts^11,12^. Therefore, the current viability of thrombectomy is largely dependent upon the use of patient selection criteria to identify patients who will see long-term benefit.

Current patient selection criteria are primarily based on inclusion criteria used in recent randomized controlled trials^7,8^. However, these criteria, which include a symptom-onset-to-groin-puncture time of less than 6 hours, are likely to be refined with future studies. One meta-analysis of these trials, for example, suggests that thrombectomy improves patient outcomes even up to a maximum of 7.3 hours from symptom onset^13^. Furthermore, the potential for improved patient selection based on acute CTP or MRI imaging has been demonstrated recently^14,15^. In particular, the DAWN and DEFUSE-3 trials found that, for patients with large ischemic penumbra on perfusion imaging, thrombectomy can be effective for up to 24 hours after symptom onset in some cases^16–18^. This suggests that image-based patient selection may allow physicians to better identify thrombectomy candidates, resulting in more treatable patients and a greater average benefit of thrombectomy.

However, visual assessment for image-based patient selection can be very challenging, while single perfusion parameter thresholding, as often conducted in the clinical setting, is not sufficient to take into account the dynamic lesion growth as well as different treatment options available. Therefore, an increasing number of multi-parametric models for final tissue outcome prediction have been proposed latetly^19–26^. The basic idea of these methods is to train high-level machine learning models based on multi-modal imaging from acute ischemic stroke patients and known follow-up outcome (*i.e*. the segmented lesion) and to use these trained machine learning models to predict voxel-wise tissue fate in new patients. In some cases, not only imaging information is used for this but also additional clinical features such as patient age and sex, time from symptom onset to treatment, and time from imaging to recanalization. By comparing predictions across multiple treatments (*i.e*. classifiers trained with data from patients treated with different treatment options), clinicians could theoretically make informed decisions regarding patient selection for thrombectomy by comparing expected tissue outcomes.

Although previous research in this domain focusses mostly on IV rtPA and rather recently also on thrombectomy as potential treatment options, clinical trials are currently investigating the benefit of additional therapeutic approaches, for example administration of NA-1 as a neuroprotective agent or hypothermia^27,28^. Therefore, treatment-specific modelling of ischemic lesion evolution for the prediction of voxel-wise tissue fate based on acute imaging is likely to become increasingly relevant to both research and clinical practice in the upcoming years as new treatment options are adopted.

This tissue outcome prediction problem was the subject of the ISLES 2017 challenge (http://www.isles-challenge.org/ISLES2017/), in which the top-performing models, convolutional and residual neural networks, employed deep learning techniques. While these novel machine learning techniques constitute a potential improvement over traditional classifiers, there is not yet consensus regarding the optimal classifier and training setup for a true comparison of novel deep learning methods with conventional machine learning techniques^29,30^. Within this context, the classification algorithm^18^, normalization of imaging features^23,24^, as well as the sampling strategy used for training sample extraction may have considerable influence on the prediction accuracy of traditional machine learning models but also partly on deep learning methods. Thus, these parameters need to be optimized for a true and fair comparison of novel deep learning approaches as well as new conventional machine learning techniques.

The aim of this study was, therefore, to compare different traditional machine learning methods, as well as different normalization and sampling techniques, regarding the ability to predict tissue outcome in acute stroke patients. Furthermore, a thresholding-based approach was evaluated to investigate the added benefit of multi-parametric tissue prediction over single-parameter thresholding.

## Materials and Methods

### Patient data

All data used in this work were acquired at the University Medical Center Hamburg-Eppendorf, Germany, between March 2003 and March 2014. All study protocols and procedures were conducted in accordance to the ethical guidelines (Ethics committee of the Hamburg Chamber of Physicians, Hamburg, Germany) and in compliance with the Declaration of Helsinki. As this was a retrospective review, the requirement of informed consent was waived. Anonymized data of 100 stroke patients with acute ischemic stroke was retrospectively collected. Inclusion criteria were as follows: (1) occlusion of either distal intracranial carotid artery or M1 segment of medial cerebral artery; (2) treatment consisting of exclusively intravenous thrombolysis therapy or in combination with intra-arterial treatment; (3) initial multi-parametric magnetic resonance imaging (MRI) including diffusion-weighted (DWI) and perfusion-weighted MRI (PWI) sequences acquired within 300 minutes of witnessed stroke symptom onset; (4) availability of follow-up MRI or computer tomographic imaging acquired 5–7 days after stroke onset; (5) absence of a preexisting proximal stenosis of the intracranial carotid artery assessed by ultrasonography, MRA, or angiographic imaging; (6) absence of an expansive and/or symptomatic intracranial hemorrhage.

### Image acquisition

For acute imaging, PWI and DWI datasets were acquired using a 1.5T Sonata or Avanto Scanner (Siemens, Erlangen, Germany). The PWI acquisition was performed after injection of approximately 15 ml contrast agent using a repetition time (TR) of 1500 ms, echo time (TE) of 45 ms, and flip angle of 90°. The parameters of the DWI acquisition were a TR = 3400 ms, TE = 100 ms, flip angle = 90°, and at least two diffusion-weightings (b-value = 0 and either 1000 or 1200 s/mm²). The DWI dataset with strong diffusion-weighting was acquired by applying diffusion gradients in three directions, which were averaged to one single strong diffusion-weighted DWI dataset. For follow-up imaging at approximately one week after stroke onset, either DWI, fluid-attenuated inversion recovery (FLAIR) MRI, or non-contrast CT (NCCT) imaging was performed.

For each patient, the NIHSS score and age at admission, sex, and time from symptom onset to imaging were also known, as well as a binarized measure of the thrombolysis in cerebral infarction (TICI) scale defining a score of >=2b as successful recanalization, or unsuccessful recanalization otherwise. Recanalization status was used to stratify the IA- and IV-treated patients (n = 50 each) into three cohorts for analysis: patients successfully recanalized using IA (IAR, n = 38), those successfully recanalized using IV rtPA (IVR, n = 23), and the remaining non-recanalizing (NR) patients (n = 39). In patients where post-treatment digital subtraction angiography was not available, recanalization was assessed using follow-up magnetic resonance angiography or computed tomography angiography.

### Preprocessing

**Figure 1:**
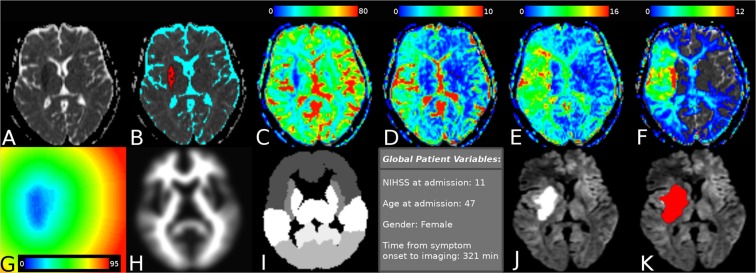
Maps of voxel features extracted in the preprocessing step. Panel (A) shows a selected ADC slice, with (**B**) superimposing the automatic segmentation of infarct core (red) and CSF (cyan). Also shown are: (**C**) CBF visualized from 0 to 80 mL/min/100 g; (**D**) CBV visualized from 0 to 10 mL/100 g; (**E**) MTT visualized from 0 to 16 seconds; (**F**) Tmax visualized from 0 to 12 seconds; (**G**) distance from infarct core visualized from 0 to 95 voxels; (**H**) the registered MNI white-matter atlas; (**I**) the discrete MNI brain atlas regions; and (**J**) follow-up imaging with the segmented real tissue outcome superimposed in (**K**).

To generate the feature datasets required for training of the tissue outcome prediction models (Fig. 1), it was necessary to extract individual features from the patient imaging data on a voxel-by-voxel level. The software tool AnToNIa was used in this work for all preprocessing steps^31^.

Briefly described, a quantitative apparent diffusion coefficient (ADC) map was calculated for each patient using the DWI datasets acquired with and without diffusion weighting. The generated ADC parameter maps were used for multiple segmentation tasks. First, the brain tissue and cerebrospinal fluid (CSF) were automatically segmented using automatic thresholding-based techniques. Only voxels belonging to brain tissue excluding CSF were included in the subsequent analyses. Next, the hemispheric fissure was manually identified to divide the ipsi- and contralateral hemisphere. Lastly, the ischemic core was semi-automatically segmented in the ADC parameter map using a volume-growing approach with manually defined seed points and an upper ADC threshold of 550 × 10^−6^ mm²/s. Manual correction in the orthogonal slices was applied in case of leakage of the region growing segmentation beyond the ischemic core. The acute diffusion lesion segmentation was then used to generate a distance map containing the shortest Euclidean distance of each voxel to the ischemic core^32^. This distance feature was included because voxels close to the acute diffusion lesion have an inherently greater risk to develop infarction compared to voxels that are very distant. Thus, the distance feature allows modelling the infarct risk depending on the distance to the acute ischemic core. However, several studies have reported partial reversal of the diffusion restriction after acute stroke reperfusion treatment so that not all voxels within the diffusion lesion can be automatically considered part of the final lesion.

In the second step, the final infarction was manually segmented in each follow-up dataset by an experienced medical expert and registered to the ADC sequence employing a rigid transformation and optimization of the mutual information similarity metric^33,34^. The resulting rigid transformation was then used to align the binary follow-up stroke segmentation to the ADC dataset applying a nearest-neighbor interpolation.

In the third step, the MNI brain atlas was registered to the patient’s ADC dataset employing an affine transformation and optimization of the mutual information similarity metric. After this, the probabilistic tissue type (white matter vs. grey matter) map defined in the MNI brain atlas as well as the MNI brain atlas regions were transformed to the ADC dataset using linear (tissue type probabilities) or nearest-neighbour interpolation (MNI brain regions). The nine MNI brain regions include the occipital, frontal, parietal, and temporal lobe as well as the cerebellum, but also important subcortical structures such as the caudate, insula, putamen, and thalamus. The idea of using these atlas regions was to include information about the localization of each voxel, as previous studies have shown that different anatomical and functional brain regions might exhibit differences regarding the vulnerability to hypoperfusion^35^. The probabilistic tissue type information was mainly expected to improve the predictive models since white and grey matter are known to exhibit significantly different perfusion values.

In the fourth step, perfusion parameter maps of cerebral blood volume (CBV), cerebral blood flow (CBF), mean transit time (MTT), and time to maximum of the residual curve (Tmax) were generated from the PWI dataset. Therefore, PWI datasets were preprocessed using an in-slice motion correction technique, slice-time correction, temporal interpolation to 1 second using a b-spline approximation, and correction to absolute contrast concentration values using the formulas described by Kjolby *et al*.^36^. Next, deconvolution-based perfusion analysis was performed using automatic atlas-based selected arterial input function definition and block-circulant singular value decomposition with a truncation threshold of 15%. After deconvolution, perfusion parameter maps were registered to the ADC dataset using a rigid transformation and optimization of the mutual information similarity metric calculated between the DWI dataset acquired without diffusion-weighting as the reference, and an average PWI dataset calculated from the first three time points of the raw PWI scan.

In addition to these voxel-level variables, four patient-level variables were added to each voxel-specific feature set through simple repetition. These were NIHSS score at admission, age at admission, sex, and time from symptom onset to imaging. As a result, each voxel was associated with a total of 12 features (Fig. 1): ADC, distance to ischemic core, tissue type, anatomical location, CBV, MTT, Tmax, CBF, NIHSS, age, sex, and time from symptom onset, whereas the real tissue outcome was used as the outcome variable for training and testing.

### Classification Methods

The desired application of machine learning models to the tissue outcome prediction problem is to predict the relative infarct risk for each voxel of an MRI volume assuming a specific stroke intervention based on the acute imaging and clinical features. Due to the large training sets resulting from the voxel-by-voxel analysis, the choice of machine learning methods is practically limited to computationally efficient algorithms, which excludes many sophisticated techniques. In this study, we compared three computationally efficient classifiers that have been used in the past for multi-parametric tissue outcome prediction^21,22,37^. Namely, the *k*-nearest-neighbor (*k*NN) algorithm, generalized linear model (GLM), and random decision forest (RDF) classifier were used. More detailed descriptions about these classifiers are given in the Supplementary Material.

### Variations of voxel-based features for tissue outcome prediction

Previous research suggests that perfusion parameters offer increased predictive power when normalized using their respective values within the contralateral hemisphere^21,23,24^. In this case, normalization may help to overcome age-dependencies and technical variations, for example, caused by the arterial input function dependence of the perfusion parameters^38,39^. Likewise, age-related variations in ADC have been described^40^ and may be accounted for through normalization of the ADC values. To investigate these assumptions in detail, instances of the three classification methods used in this work were not only trained and evaluated using absolute values of ADC and perfusion parameters but also using the corresponding values after normalization.

Two different normalization strategies were used for this purpose. In the first case, average values of ADC, CBV, CBF, MTT, and Tmax were estimated using the complete contralateral hemisphere excluding CSF voxels. In the second case, a reference volume-of-interest that roughly matches the acute diffusion lesion in the contralateral hemisphere was manually defined and used for average calculation. In both cases, ipsilateral ADC, CBF, and CBV values were normalized by calculating the ratio, while the temporal parameters MTT and Tmax were normalized by subtraction of the contralateral average values.

The final infarction typically only occupies a small fraction of the total ipsilateral brain volume. Thus, the corresponding training sets are highly unbalanced if the whole ipsilateral hemisphere or even the complete brain tissue is used for training set definition, with non-infarct voxels greatly outnumbering infarct voxels. It should be highlighted that it is usually suitable to focus on the ipsilateral hemisphere for this purpose as bi-lateral strokes are rather uncommon and non-infarct voxels significantly outnumber infarct voxels, even in most large strokes. This class imbalance can potentially bias classifiers towards under-prediction of final infarction. To evaluate a potential benefit of balanced training sets, additional training sets were generated using stratified random sampling to limit the quantity of voxels from the ipsilateral brain parenchyma (not including the follow-up lesion) to match that of the follow-up lesion for each patient. Both subsampled and full-voxel training sets were generated for each of the three normalization methods examined (non-normalized, relative to contralateral hemisphere, and relative to contralateral VOI), resulting in a total of six training sets used for training and evaluation of the three classifiers.

### Optimization and evaluation of the lesion outcome prediction models

To enable comparisons of the three classifiers, three normalization methods, and two balancing methods examined, 3 × 3 × 2 = 18 tissue outcome predictions were calculated for each patient, whereas separate models were generated and evaluated for each cohort: IAR, IVR, and NR. The advantage of training three models compared to training a single model using the cohort information as a patient-level variable, is that separate models trained on non-overlapping datasets are guaranteed to produce independent predictions. In contrast, a single machine learning model using the cohort information as a feature could result in lesion predictions that might heavily depend on training information from patients from different cohorts as this logical dependency might be missed by the machine learning model during training, which could introduce a significant bias.

For evaluation and comparison of the different machine learning models, a leave-one-patient-out cross-validation approach was used. Using this scheme, the lesion outcome was predicted for each patient using all 18 classifier-normalization-balancing combinations, resulting in a total of 1620 tissue predictions.

In all cases, the resulting tissue outcome predictions assigned a single value in the continuous range [0,1] to each voxel in the original dataset. These values represent the likelihood of the corresponding voxel to develop infarction at time of follow-up imaging, with 1 indicating the highest relative risk and 0 the lowest. Likelihood values, being a relative measure, have a distribution dependency based on the exact parameters chosen for classifier training. As a result, these values are only directly comparable for predictions within a single classifier-normalization-balancing group. To make theoretically sound comparisons between groups, it is necessary to produce an absolute measure of tissue outcome from relative likelihood values. The final threshold for each classifier setting (classifier, normalization, and subsampling) and cross validation iteration was chosen to be the threshold that produced the highest average Dice score when applied to all other patients (out-of-sample-sample) in a leave-one-out fashion. More precisely, all prediction maps for one classifier setup except the dataset used for prediction evaluation were thresholded at intervals of 0.01 over the whole range [0,1]. After thresholding, each binary prediction map was post-processed to reduce the number of misclassifications resulting from noise by applying morphological closing using a 3 × 3 × 3 voxel kernel and connected-component analysis to exclude components with less than ten voxels, which was empirically selected.

**Figure 2:**
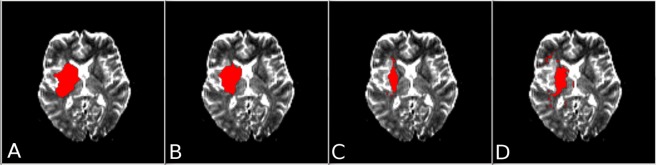
Selected slice from the ADC parameter map of a patient treated using IV rtPA with overlay showing the ground-truth lesion segmentation (**A**) and the thresholded tissue outcome predictions of: the random decision forest classifier (**B**), the generalized linear model (**C**), and the k-nearest-neighbor classifier (**D**). Training sets used by the classifiers in generating these predictions incorporated voxel subsampling and normalization relative to the contralateral hemisphere.

To correct for changes in CSF distribution due to swelling, CSF segmented in the acute ADC was excluded from the follow-up lesion. Then, for each threshold and classifier setup (Fig. 2), the Dice score (D) was calculated by comparing the binarized and post-processed predicted lesion (A) with the true follow-up lesion (B):

$$D(A,B)=\frac{2\cdot |A\cap B|}{|A|+|B|}$$

The threshold corresponding to the highest average Dice value for all training cases was then used to binarize the prediction map for the test case. Thus, the actual threshold used for binarization for each patient case within one classifier setup can vary between the datasets due to this leave-one-out approach. After thresholding, the same post-processing (morphological filtering and connected component analysis) was applied to the patient to be predicted prior to Dice calculation for evaluation.

The Dice similarity metric was the primary outcome variable as this approach finally allows calculating and visualizing detailed lesion volumes and is one of the main metrics in similar image segmentation challenges^37^. To corroborate our findings with those from other studies, secondary outcome variables including the sensitivity, specificity, Matthews correlation coefficient, mean surface distance, Hausdorff distance, and volume difference were also calculated and are reported as secondary similarity metrics.

### Evaluation of single-parameter thresholding-based prediction models

To evaluate the benefit of multi-parametric over single-parameter tissue outcome prediction, absolute as well as normalized perfusion and diffusion parameter maps of ADC, CBF, CBV, MTT, and Tmax were thresholded and compared to the ground truth using the Dice metric and the same post-processing procedure described above (optimal thresholding, morphological closing, and connected-component analysis).

### Statistical analysis

The frequency of successful recanalization following IA versus IV treatment was compared using Pearson’s Chi-squared. The same test was used to compare the sex distribution within the IAR, IVR, and NR cohorts. Additionally, the three cohorts were compared regarding the acute stroke lesion volume, penumbral tissue volume, follow-up lesion volume, age, and symptom-onset-to-imaging time using one-way ANOVAs. NIHSS at admission was compared between cohorts using the Kruskal-Wallis H test.

The Dice scores of the multi-parametric tissue outcome predictions were compared using a mixed factorial ANOVA with a between-subjects factor of recanalization cohort (IAR, IVR, NR), and within-subject factors of classifier (*k*NN, GLM, RDF), normalization (absolute, contralateral, VOI), and subsampling (none, random stratified undersampling). Similarly, the Dice scores of the thresholded single-parameter maps were compared using a mixed factorial ANOVA with a between-subjects factor of recanalization group (IAR, IVR, NR) and within-subjects factors of normalization (absolute, contralateral, VOI) and imaging parameter (ADC, CBF, CBV, MTT, and Tmax). Both ANOVAs were preceded by Mauchley’s test of sphericity and were subsequently corrected using the Greenhouse-Geisser method in all cases where sphericity could not be assumed. The results of all significant omnibus tests were further characterized using post-hoc Bonferroni-corrected pair-wise comparisons. Finally, bivariate correlation analysis was performed between the acute lesion volume and the output of the leading model configuration. All statistical tests used an alpha of 0.05 and were computed using IBM SPSS Statistics (Version 24.0, IBM, Armonk, NY).

## Results

### Patient characteristics

In total, 100 clinical datasets of acute ischemic stroke patients – 38 recanalized with intraarterial mechanical thrombectomy (IAR), 23 recanalized with intravenous rtPA (IVR), and 39 non-recanalizing (NR) – were available for this study. Five IAR datasets and five NR datasets had to be excluded from final analysis due to severe motion or imaging artifacts. The patient and imaging characteristics for the three groups are given in Table 1. The acute stroke lesion volume, penumbral tissue volume, follow-up lesion volume, age, sex distribution, and NIHSS scores at admission did not significantly differ between the patient groups. However, the symptom-onset-to-imaging time was significantly different between the patient groups, *F*(2,87) = 3.827, *p* = 0.026, with NR patients having a significantly longer onset-to-imaging time (mean M = 171.76, standard error SE = 22.08 minutes) than IVR patients (M = 97.83, SE = 12.28 minutes), *p* = 0.032. Successful recanalization (TICI>=2b) rates were significantly higher for patients treated with IA (79%) than patients treated with IV alone (48%), *p* = 0.003.

**Table 1:** Stratified mean values (and standard deviations) of patient and imaging characteristics for IAR (n = 33), IVR (n = 23) and NR (n = 34) patients.

	ADC Lesion Volume (mL)	Tissue-At-Risk Volume (mL)	Follow-up Lesion Volume (mL)	Age (years)	Percent Female	Initial NIHSS	Onset to Imaging (minutes)
IAR	19.65 (5.08)	17.58 (5.32)	50.85 (14.11)	70 (1.76)	52	15.85 (0.91)	162.61 (17.71)
IVR	7.50 (2.09)	27.9 (6.11)	32.22 (6.71)	74 (2.74)	78	14.30 (1.31)	97.83 (12.28)
NR	14.28 (3.33)	21.71 (4.72)	68.73 (12.1)	75 (2.19)	96	15.85 (0.89)	171.76 (22.08)
significance	*p* = 0.131	*p* = 0.430	*p* = 0.138	*p* = 0.086	*p* = 0.255	*p* = 0.524	***p*** = **0.026**

*p* values were generated using the following tests: for sex frequency (Percent Female), Pearson’s Chi-squared; for initial NIHSS, Kruskal-Wallis H test; for ADC lesion volume, tissue-at-risk volume, follow-up lesion volume, age, and symptom-onset-to-imaging time, one-way ANOVA.

### Multi-parametric tissue outcome prediction

Table 2 shows the results of the evaluation of all machine learning models using the primary outcome Dice similarity metric. The first significant main effect indicated was that of classifier choice, *F*(1.854,161.281) = 20.032, *p* < 0.001. Post-hoc analysis indicated that the random forest classifier (mean Dice M = 0.447, standard error SE = 0.026) outperformed the generalized linear model (M = 0.396, SE = 0.023), *p* = 0.001, and the k-nearest-neighbor model (M = 0.361, SE = 0.022), *p* < 0.001. The generalized linear model also achieved significantly higher Dice scores compared to the k-nearest-neighbor model (*p* = 0.013).

**Table 2:** Mean Dice scores and (standard deviations) for the comparisons of ground-truths in IAR (n = 33), IVR (n = 23), and NR (n = 34) patients with tissue outcome predictions.

Cohort	Model	All voxels			Stratified Random Sampling		
		Absolute	Contralateral	VOI	Absolute	Contralateral	VOI
IAR	RF	0.399 (0.267)	0.382 (0.267)	0.376 (0.271)	0.445 (0.231)	0.450 (0.233)	**0.456** (**0.231)**
	GLM	0.320 (0.244)	0.305 (0.243)	0.319 (0.241)	0.350 (0.198)	0.346 (0.193)	0.360 (0.204)
	KNN	0.318 (0.234)	0.304 (0.226)	0.299 (0.219)	0.362 (0.221)	0.317 (0.190)	0.326 (0.194)
IVR	RF	0.420 (0.249)	0.430 (0.245)	0.428 (0.237)	0.455 (0.256)	0.453 (0.254)	**0.463** (**0.255)**
	GLM	0.405 (0.236)	0.426 (0.239)	0.430 (0.241)	0.407 (0.241)	0.419 (0.243)	0.415 (0.240)
	KNN	0.399 (0.231)	0.343 (0.247)	0.343 (0.247)	0.397 (0.238)	0.364 (0.227)	0.378 (0.229)
NR	RF	0.469 (0.236)	0.491 (0.235)	0.480 (0.239)	0.481 (0.243)	**0.495** (**0.244)**	0.473 (0.251)
	GLM	0.432 (0.213)	0.472 (0.208)	0.432 (0.213)	0.418 (0.241)	0.446 (0.235)	0.423 (0.227)
	KNN	0.367 (0.267)	0.390 (0.222)	0.372 (0.223)	0.430 (0.226)	0.402 (0.228)	0.379 (0.225)

IAR = Patients recanalized using IA thrombectomy, IVR = Patients recanalized using IV tPA, NR = Non-recanalizing patients, RF = random forest, GLM = generalized linear model, kNN = K-nearest-neighbor. The highest Dice values achieved for each cohort are shown in bold.

A significant interaction was found between the classifier model and normalization method, *F*(1.779, 154.759) = 4.411, *p* = 0.017, which introduced a single exception to the previous results: only when the training data was not normalized, the k-nearest-neighbor model generated lesion outcome predictions with similar Dice values compared to the generalized linear model. Although neither normalization nor recanalization group (IAR/IVR/NR) had a significant main effect, there was a significant interaction between them, *F*(3.409,148.295) = 3.216, *p* = 0.02. Post-hoc analysis indicated that normalization to the contralateral hemisphere led to higher Dice scores compared to normalization to a contralateral VOI only in non-recanalizing patients, *p* < 0.001, while the choice of normalization method did not lead to significantly different Dice values otherwise.

With regard to the balancing method, training sets assembled using stratified random undersampling (M = 0.411, SE = 0.023) led to lesion outcome predictions with significantly higher Dice scores than training sets incorporating all ipsilateral voxels (M = 0.391, SE = 0.022), *F*(1,87) = 5.894, *p* = 0.017.

In summary, the conditions that produced the highest average Dice score incorporated the random forest classifier and stratified random undersampling, while the normalization scheme was statistically irrelevant for this classification model. The overall mean Dice score for patients analysed in this way was 0.464 (SD = 0.240) for all patients (IAR, IVR, and NR pooled). For the same group of patients, the mean accuracy was 0.994 (SD = 0.006), the mean sensitivity was 0.544 (SD = 0.279), the average specificity was 0.997 (SD = 0.003), the mean Matthews correlation coefficient (MCC) was 0.508 (SD = 0.229), the average mean surface distance (MSD) was 4.15 mm (SD = 4.39), and the mean Hausdorff distance (HD) was 19.28 mm (SD = 12.71). Bivariate correlation analysis of the results for this combination of conditions indicated that the acute lesion volume and Dice score were positively correlated with a Spearman’s rho of 0.496 (*p* < 0.001).

These quantitative results of this classifier setting were consistent when the leave-one-out evaluation was conducted again with random stratified sampling to select different voxels for training set generation. Comparing the quantitative results using three different training sets generated by random stratified sampling provided relative differences from the mean of: Dice: 0.37%, accuracy: <0.01%, sensitivity: 0.41%, specificity: <0.01%, MCC: 0.25%, MSD: 1.23%, HD: 0.58%.

### Single parameter tissue outcome predictions

**Figure 3:**
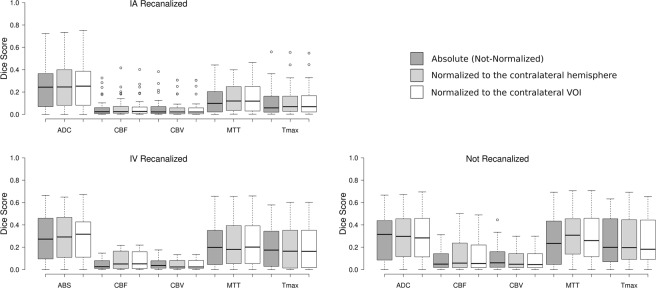
Box plots showing the distribution of Dice scores obtained from thresholded single-parameter maps of IA-recanalized (n = 33), IV-recanalized (n = 23), and non-recanalized (n = 34) patients for each normalization and treatment method. Whiskers extend 1.5 times the IQR from the 1^st^ and 3^rd^ quartile. (RC = relative to the contralateral hemisphere, RVO = relative to the VOI, ABS = absolute set of values without normalization). Unfilled dots represent outlier cases with a Dice score at least 1.5 times the interquartile range greater than the third quartile.

Figure 3 shows the mean Dice scores resulting from comparisons of the thresholded maps of individual imaging parameters (ADC, CBF, CBV, MTT, Tmax) to the ground truth. Both, the recanalization method and normalization method, were found to have significant interactions with imaging parameter, which itself had a significant main effect, F(4,348) = 4.32,*p* = 0.002.

Post-hoc analysis established a general ranking of individual imaging parameters with respect to the Dice scores. The best predictor of final infarction was ADC (M = 0.286, SE = 0.023). The other imaging parameters, in order of decreasing mean Dice score, were MTT (M = 0.220, SE = 0.019), Tmax (M = 0.192, SE = 0.019), CBF (M = 0.084, SE = 0.010), and CBV (M = 0.067, SE = 0.009). The marginal mean Dice score of each imaging parameter was significantly different from that of every other parameter with *p* < 0.001, except for MTT, which was only significantly different from ADC with *p* = 0.023 and from Tmax with *p* = 0.030. Analysis of the interaction terms relating the choice of imaging parameter to recanalization and normalization method identified some exceptions to the general ranking. When only IAR or non-normalized analyses were considered, no significant difference was found between MTT and Tmax, and no significant difference was found between CBF and CBV. Even in these cases, MTT and Tmax were still found to be superior to CBF and CBV for predicting the follow-up lesion.

The recanalization method was found to interact significantly with the choice of imaging parameter, *F*(4.125,18.130) = 2.830, *p* = 0.025. Post-hoc analysis suggested that the mean Dice score among NR patients was significantly higher than in IAR patients, but only for thresholding of the perfusion parameters MTT and Tmax, *p* = 0.005. A significant interaction was also found between the normalization method and choice of imaging parameter, *F*(3.301,11.719) = 6.786, *p* < 0.001. Mean Dice values from thresholded maps of absolute ADC and CBF were improved by using either of the normalization methods, which did not differ significantly from each other. MTT also benefitted from normalization, but only if normalized using the complete contralateral hemisphere. In contrast, absolute CBV values were more accurate than those normalized to a contralateral VOI (Table 3).

**Table 3:** Mean Dice scores (and standard deviation) for each imaging parameter and normalization method.

	Absolute	Contralateral	VOI
ADC	0.275 (0.214)	0.298 (0.216)	0.292 (0.211)
CBF	0.068 (0.079)	0.096 (0.112)	0.093 (0.112)
CBV	0.076 (0.091)	0.067 (0.079)	0.066 (0.078)
MTT	0.207 (0.188)	0.229 (0.193)	0.225 (0.196)
Tmax	0.190 (0.186)	0.193 (0.187)	0.193 (0.186)

## Discussion

### Classifier

The main result with respect to the classifier choice was that the random forest classifier outperformed the generalized linear model, which in turn outperformed the k-nearest neighbor model (Fig. 2 shows a selected single slice comparing the tissue outcome predictions of the three models). These results are generally concordant with the published results of the 2015 ISLES acute stroke penumbra estimation (SPES) challenge, which ranked variations of the random forest approach as the three best classifiers, followed by a modelling approach in fourth place, and a rule-based approach in fifth^37^. Although the results of the current study agree well with the ISLES SPES results, it should be noted that this ISLES benchmark study only evaluated machine learning classifiers for segmenting the penumbra based on multi-parametric MRI datasets, while no prediction of final infarction based on acute imaging was performed, which limits the comparison of the results.

More recently, variations of the random forest model have been developed specifically for tissue outcome prediction in acute ischemic stroke patients. The FASTER and Boosted Tree models have both outperformed generalized linear models^25,26^. The relative performance of different random forest variants, however, cannot be compared from the existing literature due to different datasets used as well as slightly different validation techniques. Regardless, these observations suggest that ensemble learning models are better suited for tissue outcome prediction than linear regression models. Furthermore, classifier comparisons performed by Gottrup *et al*. suggest that the *k*NN algorithm performs best among common instance-based methods for tissue outcome prediction^21^. The finding that the *k*NN algorithm was outperformed in the current study by both the RDF and GLM suggests that ensemble learning methods are generally better suited for the tissue prediction problem than instance-based methods.

Recently, novel deep convolutional neural network approaches are used more and more frequently for various image segmentation tasks, often yielding superior results compared to traditional image processing and machine learning techniques. These methods have also been applied for prediction of tissue outcome in acute ischemic stroke patients^41,42^. However, these studies almost exclusively use generalized linear models as a comparison method, which according to the results of this study is not the optimal conventional machine learning approach to show improved performance. Likewise, for the 2017 ISLES benchmark challenge, which compared state-of-the-art tissue outcome prediction methods, all of the submitted methods employed convolutional neural networks (http://www.isles-challenge.org/ISLES2017/), so that no comparison to optimized conventional machine learning methods could be made. This creates a demand for a well validated optimally performing conventional machine learning approach for tissue outcome prediction that can be used as a baseline to show the superiority of deep convolutional neural networks for this problem. Based on the current results, this comparison method should be a variant of the random forest classifier as opposed to the rather more common GLM.

### Normalization for classifier training

Despite the benefits of parameter normalization for training the GLM classifier demonstrated previously by Kidwell *et al*.^23^, our results showed no significant effect of normalization for the GLM classifier. Discrepancies between our findings regarding the benefit of normalization for the GLM and that done by Kidwell *et al*. may originate from application of different perfusion analysis methods. It is well known that the arterial input function selection can have significant impact on perfusion measurements. While it is not fully clear how the arterial input function was selected in the study by Kidwell *et al*., the automatic atlas-based arterial input function used in this work might lead to reduced variability of the perfusion values due to geometrically correct averaging of many concentration time curves derived from the ICA and MCA.

### Balancing method (subsampling)

The observed effect of training set balancing in the current experiment as well as previous literature suggests that machine learning classifiers perform sub-optimally on imbalanced data^43^. In the context of the tissue outcome prediction problem, a balanced training set is best achieved by using equal proportions of infarct and non-infarct voxels^43^. However, this does not imply that stratified random sampling is the optimal method for resolving data imbalance. At least for the GLM, literature suggests that 60% of the non-infarct voxels should be sampled from the penumbra and only 40% from the benign oligemia^44^. Considering the relatively small volume of the non-infarcting penumbra compared to the surrounding unaffected tissue, it is extremely unlikely that random sampling would produce this ideal distribution. While our approach used undersampling, several other techniques have been developed to mitigate the risk of introducing a bias through sampling^45^. The FASTER model, for example, randomly samples at the patient as well as voxel level, providing the major benefits of a cluster-based sampling method^27,45^. A potential future direction of study would be the characterization of specialized sampling methods such as those discussed in the recent literature^27,44^.

### Single-parameter maps

Traditionally, the core of the lesion, representing the ischemic tissue at the time of imaging, is estimated from ADC maps derived from DWI imaging^46–50^. However, there is some concern that this approach may lead to overestimation of the true ischemic core, as some parts of lesions with ADC values as low as 60% of normal have been observed to reverse in the subacute stage^51^. To the same effect, ADC has been shown to be highly time-dependent even for acute and hyperacute stroke^52^. This suggests that ADC communicates tissue viability outside the core to some extent^53^. The sensitivity of ADC to both core and penumbral tissue is one potential explanation for the unsurprising result that the best individual parameter for final infarction was found to be ADC. Furthermore, literature suggests that ADC best predicts tissue infarction within the penumbra when it is normalized to the contralateral hemisphere, which is consistent with our observation that thresholded ADC maps are better predictors of tissue fate after contralateral or VOI normalization^54,55^.

The boundary of the ischemic penumbra is typically considered the area of reduced CBF that, through compensatory vasodilation, does not have markedly reduced CBV^56^. CBF determined from PWI-MRI, however, is generally not considered a good estimator of the penumbra due to large differences in infarction thresholds for white and gray matter^57^ as well as contrast agent delay artifacts introduced by the popular standard singular value decomposition method^58^. In practice, the parameter Tmax, which benefits from increased homogeneity across tissue types^39,59^, has provided significantly more accurate predictions of the penumbra than MRI-derived CBF^60^. This is consistent with the current finding that Tmax outperforms CBF for thesholding-based lesion prediction. However, Tmax has also previously been found to outperform MTT, which we found to be the single most predictive imaging parameter^60,61^. This discrepancy may owe to the use of the block-circulant singular value decomposition method, which is delay-insensitive^62^. This interpretation is supported by additional studies showing that block-circulant singular value decomposition improves the predictive value of MTT compared to standard singular value decomposition^60^ even to the point of outperforming Tmax^63^. While CBV may have utility in multi-parametric datasets, previous studies agree that CBV alone, also when derived from CT imaging, correlates poorly with final tissue outcome^64^. In addition to suffering from heterogeneity across tissue types, CBV is often simultaneously reduced in the ischemic core and increased in the penumbra because of compensatory vasodilation and other factors^64^. Thus, a single threshold (or any linear analysis method) is often insufficient to capture both the core and penumbra, which are both relevant for final tissue outcome.

Overall, the current ranking of imaging parameters: ADC > Tmax/MTT > CBF/CBV is consistent with previous tissue prediction studies, providing some idea of the salience of these parameters as features for tissue prediction^65^. Furthermore, while no classifier benefitted from normalization of the entire multi-parametric dataset, a potential benefit of normalization was shown for single parameter ADC, CBF, and MTT thresholding, suggesting that a mixed dataset of normalized and non-normalized parameters should also be examined in a future study. Finally, it should be highlighted that the importance of the single parameters and interactions, which also include the global parameters or atlas features, for the multi-parametric lesion outcome prediction was not formally investigated for the different machine learning models evaluated here. It would be interesting to investigate if the influence of the single parameters and strength of interaction for lesion outcome prediction varies with time from stroke onset or time from imaging to recanalization. However, this is out of the scope for this work but will be the subject of a secondary study.

### Single parameter vs. multi-parametric tissue outcome prediction

Comparing the threshold-based predictions to those generated by classifiers, it is noteworthy that the Dice scores produced by single-parameter thresholding were significantly lower than those produced in the worst-case scenario by the random forest classifier. This finding confirms that multi-parametric analysis using machine learning conveys a real and significant advantage over single-parameter thresholding.

### Recanalization group

While successful recanalization was found to be significantly more common among patients treated with mechanical thrombectomy compared to IV tPA alone, the recanalization group (IAR/IVR/NR) had no significant impact on the accuracy of tissue outcome predictions. Initially, this observation seems counterintuitive, especially considering the single-parameter thresholding results that showed higher prediction accuracies for the NR group for the MTT and Tmax parameters. This is an expected finding since the lesion outcome in the NR group is the worst-case scenario and, the parameters MTT and Tmax are commonly used for estimating the penumbra or tissue-at-risk. In contrast, ADC and CBV, which were equally accurate for all patients, are mostly used for ischemic core estimation. The ischemic core is expected to remain mostly infarcted on follow-up imaging regardless of the treatment. However, the ischemic penumbra is partially salvageable assuming early reperfusion. These results suggest that tissue outcome predictions should be more accurate for the NR cohort, in which reperfusion is minimal and MTT/Tmax are highly salient, than for the IAR cohort, in which the extent of reperfusion, and therefore penumbral tissue salvage, is highly variable. The fact that such a trend was not observed speaks to the utility of training multi-parametric classifiers separately on IAR, IVR, and NR patients, but also including the time from symptom onset for the prediction. Adding temporal information may improve the lesion outcome prediction, especially for the time-dependent interpretation of MTT and Tmax perfusion maps for patients with successful reperfusion. It should be noted that only the time from symptom onset to imaging was used in this work as a temporal parameter. Additionally, the time to reperfusion is likely to be also an important parameter for tissue outcome prediction but is only available for the IAR group. Therefore, it might be valuable to include this parameter if tissue outcome predictions are only of interest for mechanical thrombectomy.

### Limitations

A few limitations of the current study need to be discussed. First, the current study was limited to patients with ICA/MCA occlusion, excluding patients with anterior circulation occlusions. The reasoning for this was to only focus on the patient cohort that is clinically relevant for decision making regarding thrombectomy.

Likewise, available follow-up imaging was limited to the period before patients were released from the hospital, 5–7 days post-ictus for practical reasons. Post-ischemic inflammation peaks between 3 and 8 days post-ictus^66,67^, leading to a possible overestimation of the final lesion volume. However, infarct volumes for a particular ischemic lesion are reported to be correlated across the acute, subacute, and chronic stage^68,69^. Therefore, while lesion volume may typically increase from the acute to the subacute stage and decrease from the subacute to chronic stage, larger acute lesions still predict proportionally larger chronic lesions, making sub-acute follow-up imaging a valid outcome variable for the tissue outcome prediction problem. Furthermore, no severe swelling or hemispheric midline shifts were observed on follow-up imaging.

While adequate for a comparative group study, it should be noted that the Dice scores achieved do not yet constitute a degree of accuracy suitable for clinical application in individual cases. This said, similar quantitative results compared to the ISLES 2017 challenge were found in this work, with the caveat that a different dataset and post-processing methods were used. Thus, the results of this work do not suggest that random forests using balanced training sets are superior or comparable to deep learning models for tissue outcome prediction but only that this setup should be used as a comparison method to prove superiority of novel methods compared to traditional machine learning models. Control over the quality and consistency of the perfusion analysis was the motivating factor behind not using the ISLES 2017 data for the current investigation as only pre-processed datasets were made available. Furthermore, it should be noted that the datasets used for ISLES 2017 included patients successfully and unsuccessfully treated with thrombectomy and thrombolysis. Thus, the 43 datasets available for classifier training is potentially not sufficient to really train three different classifiers to predict treatment outcomes individually for the two treatment types and general treatment success.

There are many potential and exciting avenues to improve the accuracy of the lesion outcome prediction. A more comprehensive discussion of external validity is limited by the choice of cross-validation over an independent validation dataset. Thus, validating the results of this study in an independent validation set, acquired in different centers, would be beneficial. However, we believe that the general results of this study hold true for the development of improved outcome prediction models, especially with respect to parameter normalization and training set balance.

More advanced approaches, such as convolutional networks, may offer improved results, but must be evaluated against a traditional classifier to quantify their superiority. The current study indicates that random forests trained on balanced multiparametric data should be used for the comparison of novel methods in this context rather than some variants of the generalized linear model.

## Conclusion

In summary, the most accurate method tested in this study, being that which employed stratified random sampling and the random forest classifier, produced an average Dice score of 0.464 while normalization of diffusion and perfusion metrics as well as recanalization success had no significant effect on the prediction accuracy.

## Supplementary information

appendix

## Data Availability

The datasets generated during and/or analysed during the current study are not publicly available due to containing information that could compromise the privacy of research participants but are available from the corresponding author on reasonable request.
