# Supplementary material for: Technical considerations of multi-parametric tissue outcome prediction methods in acute ischemic stroke patients
Source: Sci Rep. 2019 Sep 13;9:13208. doi: 10.1038/s41598-019-49460-y (PMC6744509; doi:10.1038/s41598-019-49460-y)
Supplement: Supplementary file 1 — appendix [file 41598_2019_49460_MOESM1_ESM.pdf]

Technical considerations of multi-parametric tissue outcome prediction methods in acute ischemic stroke patients

Appendix: Detailed Description of Classification Methods

Anthony J. Winder[1], Susanne Siemonsen[2], Fabian Flottmann[2], Götz Thomalla[3], Jens Fiehler[2], Nils D. Forkert[1,4,5,6]

[1]Department of Radiology, University of Calgary, Calgary, Canada
[2]Department of Diagnostic and Interventional Neuroradiology, University Medical Center Hamburg-Eppendorf, Germany
[3]Department of Neurology, University Medical Center Hamburg-Eppendorf, Germany
[4]Department of Clinical Neurosciences, University of Calgary, Calgary, Canada
[5]Hotchkiss Brain Institute, University of Calgary, Calgary, Canada
[6]Alberta Children's Hospital Research Institute, University of Calgary, Calgary, Canada

Detailed Description of Classification Methods

Machine learning classifiers are algorithms that accept the description of an item and then, through a logical analysis of its description, categorize the item. Item descriptions consist of a set of independent quantitative observations called features. The item's category is known as its class, and it is selected from a pre-defined list of discrete possibilities. The relationship between an item's features and its class is inferred by the machine learning classifier from an initial set of items for which the desired class of each item is known in addition to its features. This set is known as the training set, and it allows the classifier to make repeated adjustments to its internal logic or function until the difference between its output and desired class of each item is minimized, giving the appearance of 'learning'.

The k-nearest-neighbor (kNN) algorithm, generalized linear model (GLM), and random decision forest (RDF) classifier used in this work to predict the tissue outcome are described in more detail in the following:

The ***k*-nearest-neighbor** (*k*NN) algorithm is a simple and yet powerful instance-based machine learning method that operates by positioning training data points within a multi-dimensional space where each feature of the data is assigned its own dimension. For classification of a new data point, the *k* closest classified data points are identified in the multi-dimensional space and used to determine the classification of the new data point, for example, applying majority voting. Thus, no actual training is required for this classification technique, which makes it a fast alternative for the goal of this research question. The number of neighbors considered for any unclassified data point is described by *k*, which can be any natural number. The concept of 'nearness' is defined mathematically by a distance function, which can vary between *k*NN implementations. The *k*NN method is very efficient in lower dimensions but is known to suffer from a significant increase in complexity when considering higher dimensions and large training sets. However, it is possible to considerably simplify the original *k*NN algorithm by approximating the identity of the *k* nearest neighbors rather than calculating them exactly. By using this approximation, the algorithm can execute several orders of magnitude faster than the basic *k*NN algorithm with little additional error. In this work, we used the *k*=100 nearest data points, the Euclidean distance as a distance function for classification, and approximation of the closest neighbours. *k*=100 was selected to obtain a likelihood value for each voxel to develop infarction (percentage of the 100 closest neighbours that developed infarction).

The **generalized linear model** (GLM) is used in situations where the behavior of a general linear model is desired, but some underlying assumptions of the general linear model are violated. Generalized linear models are used when the range of the response variable is restricted and when the response variable follows a non-normal distribution. The generalized linear model calculates a quantity η that is a linear combination of independent predictor variables. For the 12 classification features that are used in this work, this can be shown as $\eta = \beta_0 + \beta_1 x_1 + \beta_2 x_2 + \cdots + \beta_{12} x_{12}$, where x_{1-12} are the 12 input features (ADC, lesion distance, tissue probability, brain region, CBF, CBV, etc.), β_0 is the regression coefficient for the intercept, and β_{1-12} are the regression coefficients that are computed from the training data. The response variable of the generalized linear model is defined as $\mu = g(n)$ where the function g, known as the link function, transforms the linear predictor to produce a response variable having the desired range and error distribution. In this work, a logistic link function $g(x) = (1 + e^{-x})^{-1}$ was used for this purpose, which constrains the range of μ to (0,1). During training, the regression coefficients β_{1-12} are optimized using the least squares method, which minimizes the error between μ and the true voxel class $c_i \in \{0,1\}$ where 0 represents unaffected tissue and 1 represents infarction. The output of the trained GLM is a value between 0 and 100% indicating the probability for the voxel to develop infarction.

The **random decision forest** (RDF) classifier is an ensemble learning classifier that integrates concepts of decision trees and bootstrap aggregation (bagging) into a classification model with distinct advantages over traditional decision tree classifiers. Traditional decision trees attempt to infer the class of novel data points by comparing them to similar pre-classified data points. In contrast to the *k*NN method, this comparison is performed using a series of attribute value tests that are learned from existing data. Decision tree classifiers typically have low bias but, having a high variance, suffer from a tendency to overfit the training data and produce classification models with reduced generalizability as a result. Random decision forests use bagging to minimize overfitting effects of single decision trees by training many classification trees on randomly sampled subsets of the training data and then aggregating their outputs. In this work, a random forest algorithm with aggregation of 100 decision trees was used. Each tree was trained from a randomly sampled set of training points equivalent to 50% of the total training data in size. The 100 decision trees are used for the same reason as *k*=100 is used for the *k*NN-based tissue outcome prediction. Thus, the RDF method will produce a likelihood value for each voxel to develop infarction that is calculated from the number of trees predicting infraction (0-100%).
